# Biology and systematics of the New World *Phyllocnistis* Zeller leafminers of the avocado genus *Persea* (Lepidoptera, Gracillariidae)

**DOI:** 10.3897/zookeys.97.753

**Published:** 2011-05-11

**Authors:** Donald R. Davis, David L. Wagner

**Affiliations:** 1 Department of Entomology, National Museum of Natural History, Smithsonian Institution, P.O. Box 37012 MRC 105, Washington, D.C. 20013–7012, U.S.A.; 2 Ecology and Evolutionary Biology, University of Connecticut, Storrs, Connecticut 06269–3043, USA

**Keywords:** Biogeography, hypermetamorphosis, genital morphology, larval morphology, pupal morphology, cocoon cutter, serpentine mine, DNA barcodes

## Abstract

Four New World species of *Phyllocnistis* Zeller are described from serpentine mines in *Persea* (Family Lauraceae). *Phyllocnistis hyperpersea*,new species, mines the upper leaf surfaces of avocado, *Persea americana* Mill., and red bay, *Persea borbonia* (L.) Spreng. and ranges over much of the southeastern United States into Central America. *Phyllocnistis subpersea*,new species, mines the underside and occasionally upper sides of new leaves of *Persea borbonia* in southeastern United States*. Phyllocnistis longipalpa*, new species, known only from southern Florida also mines the undersides of new leaves of *Persea borbonia*. *Phyllocnistis perseafolia*,new species, mines both leaf surfaces and possibly fruits of *Persea americana* in Colombia, South America. As in all known species of *Phyllocnistis*, the early instars are subepidermal sapfeeders in young (not fully hardened) foliage, and the final instar is an extremely specialized, nonfeeding larval form, whose primary function is to spin the silken cocoon, at the mine terminus, prior to pupation. Early stages are illustrated and described for three of the species. The unusual morphology of the pupae, particularly the frontal process of the head, is shown to be one of the most useful morphological sources of diagnostic characters for species identification of *Phyllocnistis*. COI barcode sequence distances are provided for the four proposed species and a fifth, undescribed species from Costa Rica.

## Introduction

**Figure 1.**
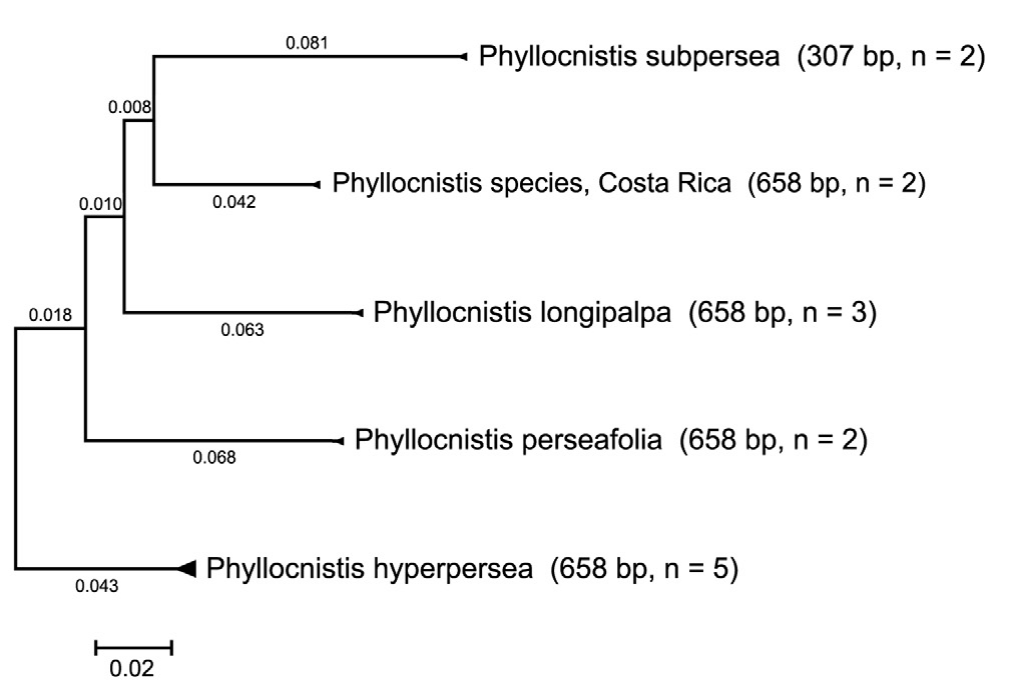
COI distance tree of five *Persea*-mining species of *Phyllocnistis* based upon neighbor-joining analysis with Kimura 2-parameter model.

Although the genus *Phyllocnistis* Zeller, 1848 is one of the more speciose genera in the Gracillariidae, its diversity is greatly underestimated in existing taxonomic literature. Currently 90 valid names are known for the world (DePrins and Kawahara 2009, De Prins and De Prins 2011), with 28 listed for the New World (Davis and Miller 1984, Kawahara et al. 2009). During 1996 – 1998, as part of the Arthropods of La Selva (ALAS) Project, the authors conducted a preliminary survey of the Gracillariidae of La Selva Biological Station, a lowland rain forest site in northwestern Costa Rica. Because our visits to the study site were limited to less than 15 days each year, our survey was restricted largely to the collection of leafmines and examination of plant specimens in the station’s herbarium. Relying primarily on mine morphology and host plant information, we estimated that as many as 200 species of Gracillariidae were present at La Selva Biological Station. By far the most speciose genus in our survey was *Phyllocnistis* with an estimated 60 species, of which we recognized only a single previously known species, the invasive citrus leafminer, *Phyllocnistis citrella* Stainton. Interestingly, this globally monitored species had been first reported in the Americas (Florida and the West Indies) only three years earlier in 1993 (Heppner 1993, Hoy and Nguyen 1997). Further, we believe it probable that several more *Persea*-feeding *Phyllocnistis* will be found considering that there are more than 200 species of *Persea* known globally (Mabberley 2008), and that as many as three species may use a single host species at a single location: e.g., *Persea borbonia* at Pa-hay-okee Overlook in the Florida Everglades. Although only four species are proposed in this report, a fifth species based on COI barcode sequences (Fig. 1) and unique pupal characters (but without properly associated adults) has been examined from *Persea americana* collected in Costa Rica. While preliminary, our studies suggest that tropical diversity of *Phyllocnistis* will someday tally in the hundreds of species.

**Figure 2.**
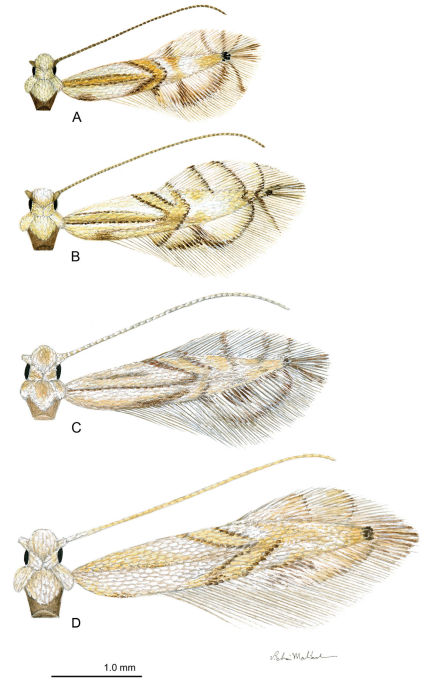
*Phyllocnistis* adults. **A** *Phyllocnistis hyperpersea* sp. n. (2.1 mm) **B** *Phyllocnistis subpersea* sp. n. (2.5 mm) **C** *Phyllocnistis longipalpa* sp. n. (2.6 mm) **D** *Phyllocnistis perseafolia* sp. n. (3.0 mm). (Drawn approximately to scale; forewing length in parentheses.)

Adults of the genus *Phyllocnistis* are very small moths with wing spans generally not exceeding 5 mm. Both fore- and hindwings are lanceolate and predominantly white; the forewings are marked with yellow to orange, longitudinal and oblique striae, often bordered by gray or black (Figs 2A–D). A few species are known to possess much darker or strikingly color patterns. Terminology used for describing forewing pattern follows that of Kawahara et al. (2009). The compound eyes of *Phyllocnistis* are reduced, with an interocular index (vertical eye diameter/minimum interocular distance) of approximately 0.9. The maxillary palpi are the most reduced among Gracillariidae, being barely evident as vestigial, non-segmented lobes at the base of the elongate proboscis. The wing venation (Forbes 1923) is also reduced with M1 stalked to Rs4; M3 and CuA2 are lost in the forewing; the base of M is absent within the forewing discal cell, and the cell is open in the hindwing. The apex of the forewing is unusual in being greatly attenuated with Rs4 extending to the apex.

The morphology of the male genitalia is relatively simple and usually characterized by a relatively broad vinculum, narrow tegumen and slender valvae, and species-level differences are modest relative to other gracillariids or Lepidoptera in general. Homology of what appears to be segment 10 is uncertain and consists of a mostly membranous cylinder extending caudally from the sclerotized tegumen that mostly encompasses the anal tube (Fig. 16A).

The female genitalia, although characteristic for the genus, likewise appears relatively uniform among species, consisting primarily of very short papillae anales, a comparatively large, oblong corpus bursae often containing a pair of similar, fusiform signa bearing a short median projection (Fig. 16D); a pair of similar, slender, elongate ducts that extend from usually opposite ends of the corpus bursae, one (ductus bursae) leading to the ostium and the other (ductus spermathecae) to the spermatheca.

The larvae of *Phyllocnistis* are among the most specialized Lepidoptera (Trägårdh 1913). Four instars appear to be the norm (Condrashoff 1962, Wagner and Davis unpublished data), with the first three instars possessing a sapfeeding morphology and behavior (Davis 1987). Sapfeeding instars create a long serpentine, subepidermal mine on either the upper or lower surfaces of the host leaf. A few species also form subepidermal mines on stems and various fruits, including avocado. A characteristic, median frass trail extends the length of the mine, usually as a dark, unbroken line. The fourth instar is a highly specialized, apodal, non-feeding instar whose primary function is to spin the cocoon, at the mine terminus, prior to pupation.

In contrast to the conservative morphology of the larval and adult stages, the pupae of *Phyllocnistis* are structurally diverse, particularly with regard to the development of the frontal process (cocoon-cutter) of the head (Figs 6A, B; 12A, B; 14A, B; see also Kawahara et al. 2009). In addition, the mid-dorsal areas of abdominal terga 3–7 possess a mostly symmetrical cluster of recurved spines that frequently differ in their arrangement and form among species. Given the uniformity in both male and female genital characters in *Phyllocnistis*, it is surprising to us to find reliable species-level differences in pupal morphology across what appear to be closely-related congeners feeding on *Persea*. We certainly encourage others to collect and illustrate pupae whenever new species are described in the genus.

Consistent differences in wing patterns were noted for each of the four species described here—although such are easily abraded if specimens are not collected and prepared with care. We note that our descriptions are based solely on reared material and thus might differ in appearance from flown specimens. Because of the general similarity of both male and female genitalia that exists among most members of *Phyllocnistis*, species identification relying upon standard genitalic characters may be impossible at times. We expect that the application of COI barcoding will be especially useful in this large genus of minute moths.

The plant genus *Persea* includes approximately 200 species worldwide, with a majority of the species concentrated in Central America and southeast Asia (Mabberley 2008); Kopp (1966) recognized 81 species for the New World. The most important species economically is the avocado, *Persea americana* Mill., which is grown throughout the tropics for its fruit.

Half of the *Phyllocnistis* found in eastern North America are hostplant specialists on archaic families of woody plants: 3 species feed on *Persea* in the Lauraceae, 2 on *Magnolia* and *Liriodendron* in the Magnoliaceae, and 1 on *Liquidambar* in the Hamamelidaceae (or Altingaceae), all plant families that date to the Cretaceous. Ninety seven-million-year-old (more recently estimated at 102 mya, Brenner et al. 2000) phyllocnistine leafmines provide the oldest fossil evidence of Ditrysian Lepidoptera (Labandeira et al. 1994). Phylogenetic studies will be needed to ascertain if the association of the genus with archaic plant families is a testament to the ancient and often conservative nature of insect-plant associations (Farrell et al. 1992, Labandeira et al. 1994, Wilf et al. 2000) or the result of more recent host colonizations.

The damage inflicted by *Phyllocnistis* larvae feeding on avocado may vary according to region and the species of miner involved. Wysoki et al. (2002) reported major damage to avocado caused by an unknown *Phyllocnistis* in Peru that could reduce tree vigor,but only minor damage on avocado by *Phyllocnistis* in Florida. Larval feeding by *Phyllocnistis perseafolia* on avocado leaves in Colombia is known to cause serious damage (Francisco Posada, *in litt*., Fig. 4). Possibly the damage to avocado in Peru reported by Wysoki et al. (2002) was also produced by *Phyllocnistis perseafolia*.

## Material

The material examined is deposited in the collections listed below:

BMNH The Natural History Museum (formerly the British Museum (Natural History)), London, United Kingdom.

FSCA Florida State Collection of Arthropods, Gainesville, Florida, USA.

UCMS University of Connecticut, Storrs, Connecticut, USA.

UNCM Museo Entomolgico Francisco Luis Gallego, Universidad Nacional de Colombia, Medellín, Colombia.

USNM Collections of the former United States National Museum, now deposited in the National Museum of Natural History, Smithsonian Institution, Washington, D.C., USA.

## Methods

Collecting and rearing.Nearly all of the adults in this study were reared from their plant hosts. Leaves containing mines with larvae were placed in plastic bags or large plastic tubes and examined daily thereafter. Newly-eclosed adults were killed with ammonium hydroxide fumes or frozen, pinned, and spread. Some larvae representing different instars and pupae were fixed in Pampel fluid and preserved in 75% ethanol. Samples of alcohol-preserved larvae and pupae were gently washed in 409® detergent, then dried in a critical point drier, sputter coated with 20–25 gold palladium 60:40 alloy, and photographed with an Amray 1810 scanning electron microscope.

Genitalic dissections were cleared by heating in hot 10% KOH for ~ 30 minutes, and subsequently cleaned and stained with either 2% chlorazol black E or mercurochrome solutions. All genitalic illustrations were drawn from dissections temporarily stored in glycerine, which were later permanently embedded in Euparal or Canada balsam. Genitalic terminology follows Klots (1970).

Molecular analysis.DNA **s**equences were produced at the Biodiversity Institute of Ontario, University of Guelph, Canada. DNA was extracted from legs or entire bodies of adult moths using a QIAGEN DNeasy Tissue Kit. Primers LepF1 and LepR1 (Herbert et al. 2004) were used to obtain a 658 base pair fragment of COI with a standard thermocycling regime (Hajibabaei et al. 2006). Sequences are available at the National Center for Biotechnology Information GenBank database and at the Barcode of Life Database (BOLD). Neighbor-joining (NJ) trees were generated from 14 nucleotide sequences as implemented in BOLD (Ratnasingham and Hebert 2007).

## Results

Exemplars of all five species clustered as each other’s closest neighbors. A compressed subtree (Fig. 1) of the COI barcode sequences from the 14 specimen samples was computed using Molecular Evolutionary Genetics Analysis (MEGA) version 4 (Tamura et al. 2007). Only partial sequence data were obtained for *Phyllocnistis subpersea*. Uncorrected pairwise distances exceeded 10% between species. *Phyllocnistis hyperpersea*, the only species in our study that preferentially mines upper leaf surfaces, clustered outside of the group that mine lower leaf surfaces.

**Table 1.** Sample information for specimens submitted for COI barcoding. Additional specimen data as well as sequence data are available on the BOLD website at RDOPO Basal Lepidoptera.

**Sample** *ID*	Species of *Phyllocnistis*	*Locality*	*BOLDProcess ID*	*GenBankAccession number*
DDAV–D557	*Phyllocnistis hyperpersea*	USA:FL: Pah-hay-okee	RDOPO395-10	HQ971045
DDAV–D558	*Phyllocnistis hyperpersea*	USA:FL: Pah-hay-okee	RDOPO396-10	HM382098
DDAV–D559	*Phyllocnistis hyperpersea*	USA:FL: Tamarind Hammock	RDOPO397-10	HQ971046
USNM ENT 00730716	*Phyllocnistis hyperpersea*	USA:FL: Tamarind Hammock	EPNG1734-10	HQ946656
USNM ENT 00730718	*Phyllocnistis hyperpersea*	USA:FL: Pah-hay-okee	EPNG1735-10	HQ946657
DDAV–D555	*Phyllocnistis perseafolia*	Colombia: Caldas	RDOPO393-10	HM382096
DDAV–D556	*Phyllocnistis perseafolia*	Colombia: Caldas	RDOPO394-10	HM382097
DDAV–D562	*Phyllocnistis longipalpa*	USA:FL: Tamarind Hammock	RDOPO400-10	HM382099
DDAV–D564	*Phyllocnistis longipalpa*	USA:FL: Cheika	RDOPO402-10	HM382100
USNM ENT 00718383	*Phyllocnistis longipalpa*	USA:FL: Pah-hay-okee	EPNG1759-10	HQ946666
DDAV–D565	species	Costa Rica: Cart.: Tres Rios	RDOPO403-10	HM382101
DDAV–D566	species	Costa Rica:	RDOPO404-10	HM382102
USNM ENT 00730717	*Phyllocnistis subpersea*	USA:FL: Tamarind Hammock	EPNG1736-10	
USNM ENT 00730756	*Phyllocnistis subpersea*	USA:FL: Pah-hay-okee	EPNG1737-10	

## Taxonomy

### Key to Adults and Pupae

**Table:** 

1	Forewing tornus with raised row of broadened, black fringe scales; labial palpus short (circa 1.3× height of eye), roughened at apex; forewing often with fuscous subbasal spot; apical spot poorly developed, never blackened; frontal process (cocoon-cutter) of pupa consisting of a pair of stout, conical spines arising near apex, and single, more subapical, strongly curved spine from upper frons (Figs 12A–C)	*Phyllocnistis subpersea*
–	Forewing tornus without distinct row of broadened, raised, black fringe scales; labial palpus short (less than height of eye) or long (circa height of head) smooth at apex; forewing without dark subbasal spot (although basal area may have fuscous scales); apical spot present or absent; frontal process of pupa a single spine (unknown for *Phyllocnistis longipalpa*)	2
2	Palpi short (less than height of eye); smaller, FW < 2.2 mm; prominent black apical spot; frontal process relatively large, broadly triangular, acute (Figs 6A–B)	*Phyllocnistis hyperpersea*
–	Palpi long (>2× height of eye), slightly upcurved; FW > 2.2 mm; apical spot present or absent	3
3	Forewing length ≤ 2.6 mm; apical spot vague; orange scales at base of wing not extending to costa; basal streak narrowly edged with black; pupa unknown. North American	*Phyllocnistis longipalpa*
–	Forewing length ≥ 2.6 mm; apical spot prominent; orange scales at base of wing reaching to costa, basal streak not edged with black; frontal process of pupa composed of single, large apical spine with minutely serrated, low ridge descending laterally from spine (Figs 14A–B). South American	*Phyllocnistis perseafolia*

### Species descriptions

#### Phyllocnistis hyperpersea

Davis and Wagner sp. n.

urn:lsid:zoobank.org:act:896DF46D-4693-4028-A909-0C9B3DC5E41C

http://species-id.net/wiki/Phyllocnistis_hyperpersea

Figs 2A 3A 5A 6A 7D 16 A–E

##### Diagnosis:

*Phyllocnistis hyperpersea* is the smallest of the species treated here with FW lengths < 2.3 mm. The short labial palpi (less than height of eye) and prominent black apical spot, taken together, distinguish *hyperpersea* from the other *Persea*-feeding *Phyllocnistis*. The second costal fascia is weakly developed and does not fuse with the transverse fascia as in other *Phyllocnistis* treated here. Hind tarsomere 3 is more likely to be black than that of the other species. The black fringe scales about the tornus are less conspicuous: fewer in number, narrower, and less blackened relative to those of *Phyllocnistis subpersea* with which it commonly co-occurs. The frontal process of the pupa extends forward as a relatively large, broadly triangular, acute spine (Figs 6A–B).

##### Adult

(Fig. 2A): Length of forewing: 1.9–2.2 mm.

*Head:* Frons shiny white, smooth glabrous, with subtle faint orange tints over vertex. Flagellomeres with orange-fuscous luster above. Labial palpus white, reduced, length less than height of eye.

*Thorax:* Patagia and tegulae with silvery stramineous to orange tints. Forewing with longitudinal fascia usually ending before joining transverse fascia, thinly edged with black scales above and below except distad. Transverse fascia usually complete, leaving costal margin at 45° angle; usually more thickly edged with black scales along proximal side; distal side somewhat rounded with black, edge-scaling weakened medially. Second costal fascia poorly differentiated, not fusing with transverse fascia. Apical spot of black scales well developed. Costal and apical strigulae modestly differentiated, often only two of latter evident. Black fringe scales about tornus only modestly differentiated: few in number, not strongly raised, and not appreciably broadened. Dorsal and outer surfaces of foretibiae and foretarsi, and to lesser extent those of mesothoracic legs, with fuscous metallic orange; third tarsomere of hindleg often darkened; otherwise legs mostly silvery white and unmarked.

*Abdomen:* Silvery white and unmarked.

*Male Genitalia* (Figs 16A–C): Uncus absent. Tegumen complex, consisting of narrow, sclerotized dorsal arch, continuing caudally as far as apex of valva as an elongate, mostly membranous, basally spinose cylinder which encloses anal tube. Vinculum well developed, ~ 0.5× length of valva, U- to V-shaped with relatively narrow anterior end. Valva simple, relatively long, ~ 2.0× length of vinculum, very slender and straight; broad at extreme base, then narrowing along middle, becoming slightly broader over apical third; apex of valva evenly rounded; basal apodeme of valva directed mesad at nearly right angle to valva. Transtilla arising from mesal base of valva as an elongate, acute process, and continuing mesally to articulate at midline with process from opposite valva. Aedeagus slender, weakly sclerotized, externally finely wrinkled cylinder ~ half length of valva; cornuti absent; phallobase greatly extended as membranous tube ~ 6× length of aedeagus; terminal hood of phallobase abruptly inflated and curved at ~ right angle to phallobase.

*Female Genitalia* (Figs 16D–E): Oviscapt greatly reduced; anterior and posterior apophyses of about equal lengths, very short, ~ 0.4× length of papillae anales. Ostium bursae opening in membrane between sterna 7 and 8; ductus bursae completely membranous, slender, moderately long, ~ 2.7× length of papillae anales and terminating near caudal third of corpus bursae; corpus bursae greatly enlarged, ~ 1.5× length of ductus bursae; walls of corpus bursae membranous except for pair of approximately identical, fusiform signa, with each bearing single inward-projecting, acute, blade-like process; length of process ~ 0. 2× length of signum; ductus seminalis extremely slender, elongate, ~ 1.3× length of corpus bursae, arising from cephalic end of corpus bursae.

##### Larva:

Sapfeeding instar (Fig. 5A): Similar to *Phyllocnistis subpersea* except: length of largest larva examined ~ 4.4 mm; labrum well developed, with lateral margins evenly rounded, not produced caudally as in *subpersea*; caudal processes of last (9+10th) abdominal segment ~ half length of entire segment.

Last instar larva not examined, but probably similar to that of *Phyllocnistis subpersea*.

##### Larval Mine

**Figure 3.**
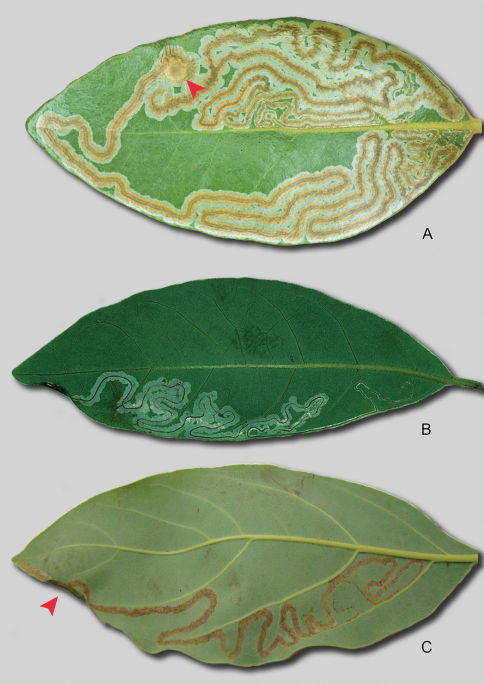
*Phyllocnistis* leafmines. **A** *Phyllocnistis hyperpersea* sp. n., upper-side mine on *Persea borbonia* **B** *Phyllocnistis subpersea* sp. n., lower side mine on *Persea borbonia*. Pupal crypts indicated by arrows in A and C. **C** *Phyllocnistis perseafolia* sp. n., lower-side mine on *Persea americana* (~ 15 cm).

(Fig. 3A): A long, slender, serpentine gallery, with a relatively broad, dark brownish, median frass trail, almost always located on the upper (adaxial) side of the leaf (only 1 under (abaxial) side mine found). The egg is deposited on the upper leaf surface away from the midrib. Mine begins on one side of the blade, but after much of one side is consumed, crosses over near the leaf apex to the other side. The median frass line is unusually broad for a species of *Phyllocnistis*, resembling more that of the Chilean genus *Prophyllocnistis* (Davis 1994). As previously noted (Davis 1994), the mines of *hyperpersea* are also similar in general morphology to early Cenomanian phyllocnistine leafmines (Labandeira et al. 1994). Pupation occurs in the lamina, away from the leaf edge (~ 5–7 mm in diameter) in a circular nidus, similar to that fashioned by *Prophyllocnistis*. The serpentine portion of the mine begins as a narrow tract ~ 0.3 mm wide and gradually enlarges before the pupation chamber to a width of ~ 2–2.5 mm. The median frass line is ¼ of the mine width in the early instars and gradually broadens to more than half the mine width.

**Figure 4.**
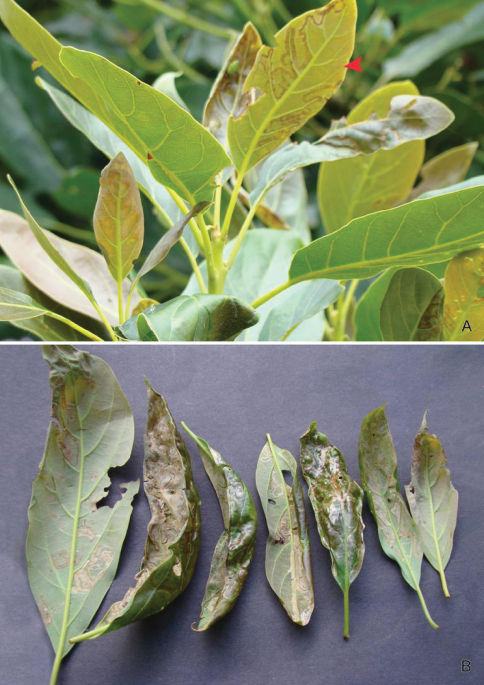
Leafmines of *Phyllocnistis perseafolia* sp. n. on *Persea americana*. **A** General habitus, note lower side mine (arrow) **B** Leaf damage caused by upper and lower side larval mining.

**Figure 5.**
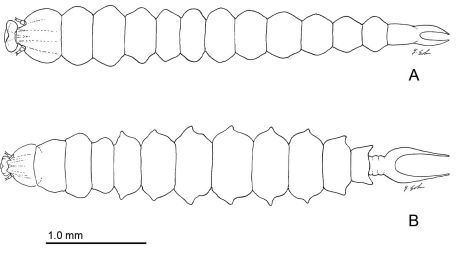
Late instar sapfeeding larvae. **A** *Phyllocnistis hyperpersea* sp. n. (length 4.3 mm) **B** *Phyllocnistis subpersea* sp. n. (length 4.4 mm).

**Figure 6.**
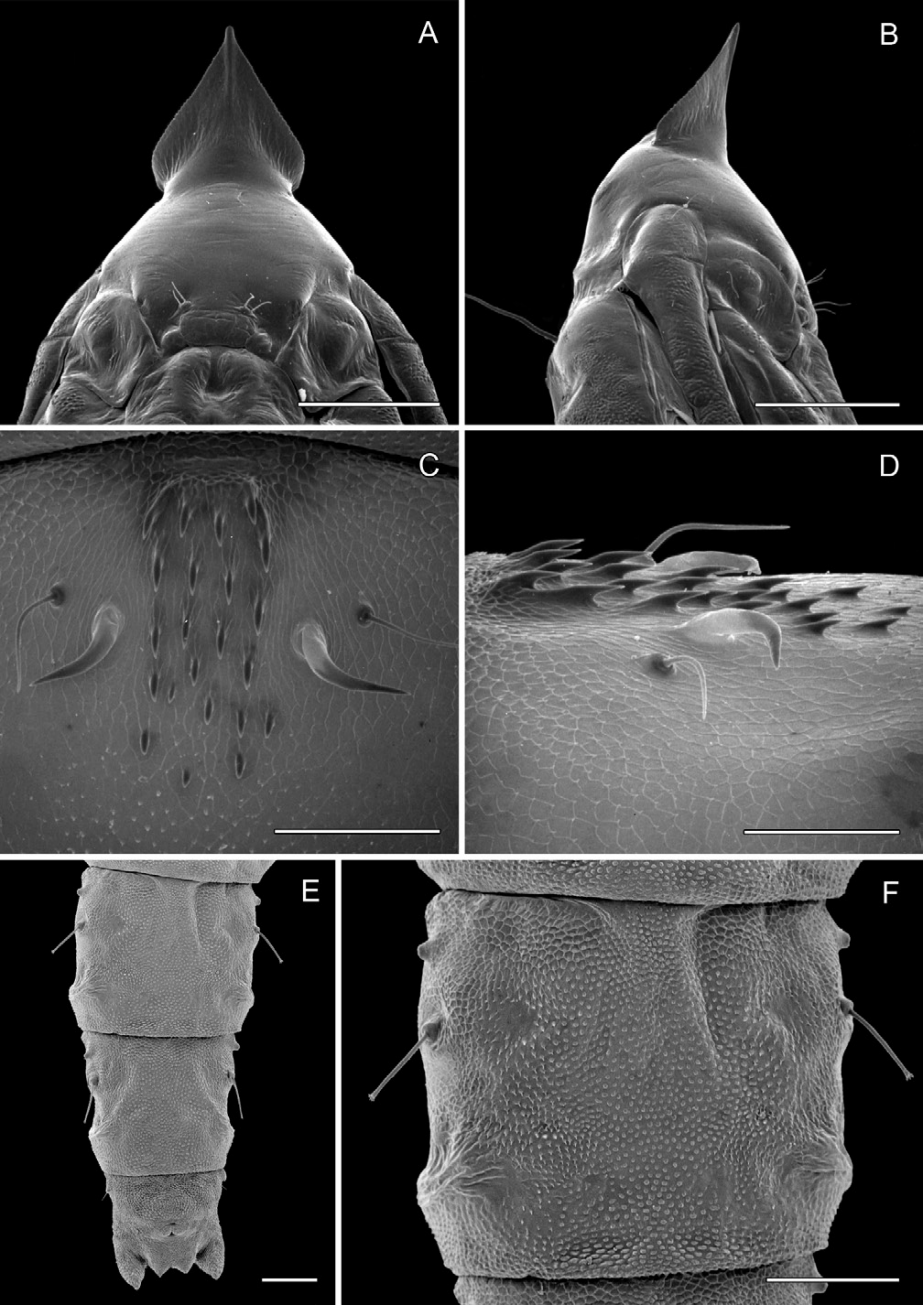
*Phyllocnistis hyperpersea* sp. n. pupa. **A** Head, ventral view (176 µm) **B** Head, lateral view (200 µm) **C** Dorsal spines of abdominal tergum 5 (76 µm) **D** Lateral view of **C** (60 µm) **E** Abdominal sterna 6–10 (100 µm) **F** Scattered spinules of sternum 6 (100 µm). (Length of bar scales shown in parentheses.)

**Figure 7.**
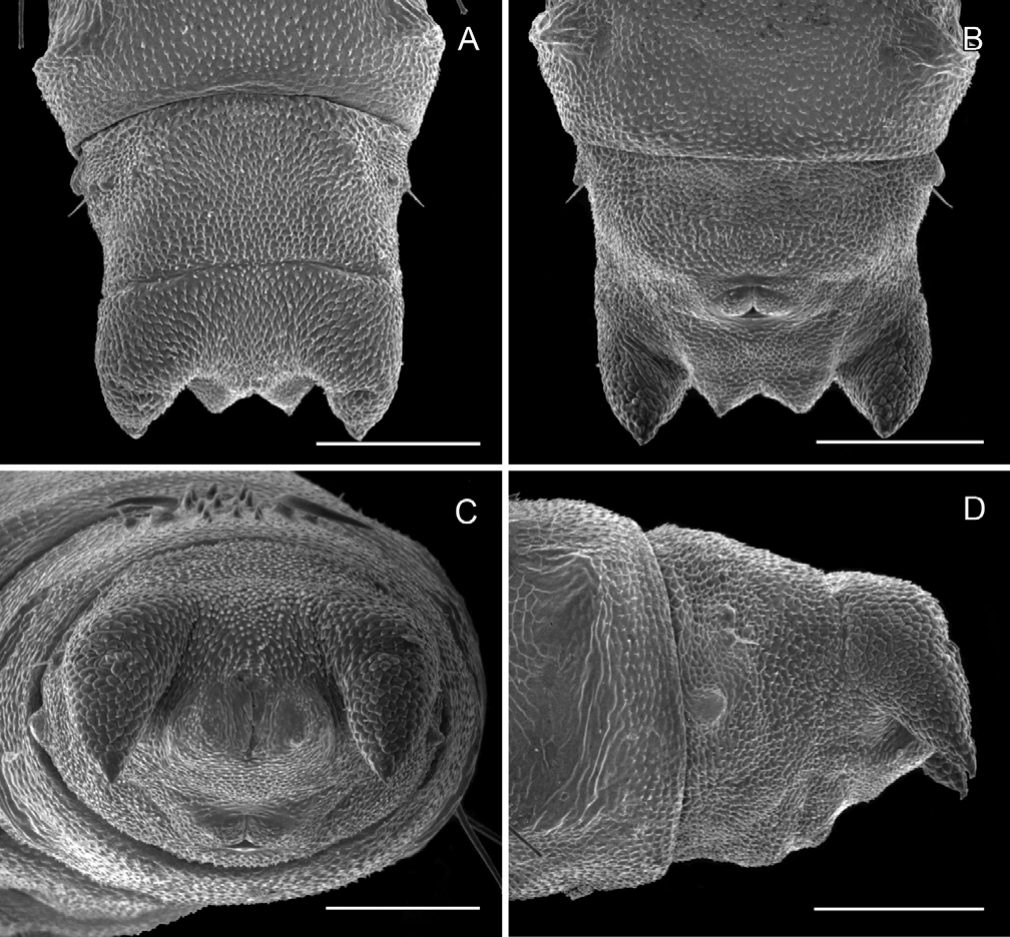
*Phyllocnistis hyperpersea* sp. n. pupa. **A** Abdominal terga 6–10 (100 µm) **B** Abdominal sterna 6–10 (100 µm) **C** Caudal end of abdomen (100 µm) **D** Lateral view of abdominal segments 6–10 (100 µm). (Length of bar scales shown in parentheses.)

**Pupa** (Figs 6A–7D): Length of largest pupa 2.8 mm, maximum diameter 0.7 mm. Vertex with relatively large, broadly triangular, acute frontal process (cocoon-cutter) similar to that of *Metriochroa psychotriella* Busck, but with base constricted slightly on each side (Fig. 6A); lower frons with 2 pairs of short frontal setae. Antenna long and straight, extending almost to 7th abdominal segment (A7); forewing extending almost to A6. Abdominal setae generally short except for greatly lengthened SD1 on A2–7; apex of SD1 on A2–7 slightly enlarged, but not spatulate; abdomen with 6 mid-dorsal pairs of spine clusters (Figs 6C–D) beginning near anterior margins of terga 2–7; each cluster with series of similar, low, strongly recurved spines arranged in 4 irregular columns of about 4–6 ranks; pair of much larger, strongly recurved spines immediately lateral to central cluster and adjacent to seta D1 on A4–7; sternum A6 with spinules evenly scattered over surface (Fig. 6F); A10 with pair of relatively large, stout, caudal projections arising laterally and directed ventrally (Figs 7A–D).

**Host**: *Persea americana* Mill., variety Blair, *Persea borbonia* (L.) Spreng.

**Type** **Material**: Holotype: ♂, USA: FLORIDA: Dade Co: Everglades National Park, Pa-hay-okee Overlook, 26°27'N, 80°47'W, 24 Nov 1991, emerged 2 Dec 1991, D. Davis, DRD 1020.1, host: *Persea borbonia*, USNM slide 31635 (USNM). Paratypes: USA: FLORIDA: Dade Co: Everglades National Park, Pa-hay-okee Overlook, 26°27'N, 80°47'W, mine 24 Jun 1990, DRD 724, host: *Persea borbonia*: 1♀ emerged 26 Jun 1990, USNM slide 31637; mines 25 Nov 1991, D. L. Wagner and D. R. Davis, DLW Lot: 91L121, host: *Persea borbonia*: 4♂, 2♀ emerged 27 Nov to 4 Dec 1991 (UCMS); mines 16 Apr 1995, D. Davis, DRD 1626.1, host: *Persea borbonia*: 7♂, 2♀ emerged 26 Apr 1995, BOLD ID: RDOPO396-09, 2♂ emerged 27 Apr 1995, 3♂, 2♀ emerged 28 Apr 1995, DRD 724, BOLD ID: RDOPO395-09, host: *Persea borbonia* (BMNH, NMNH). Dade Co: Everglades National Park, Long Pine Key, 26°24'N, 80°41'W, mines 21 Feb 1992, D. and S. Davis, DRD 1060, host: *Persea borbonia*: 1♀, emerged 27 Feb 1992, slide USNM 31636 (USNM). Homestead: 1♂, 1♀ 1 Sep 1993, R. E. Duncan, J. E. Pena, M. Biondo, host: *Persea americana*, (USNM); 1♂, 1 May 2008, J. E. Pena, 08-2811, 4 upperside leafmines, 2 pupae, host: avocado (FSCA). Highlands Co: Archbold Biological Station, 11 km S. Lake Placid: 8 leafmines, 14 Jun 1992, DRD 1097, host: *Persea borbonia* (USNM); Highland Hammock State Park: 7 leafmines, 15 Jun 1992, DRD 1097.1, host: *Persea borbonia* (USNM). Liberty Co: Appalachicola National Forest: 2 leafmines, 18 Jun 1992, DRD 1097.2, host: *Persea borbonia* (USNM). Monroe Co: Big Cypress National Preserve: Loop Road near Tamarind Hammock, 26°27'N, 80°31'30"W: mines 13 Mar 1991, D. L. Wagner and D. R. Davis, DLW Lot: 91C138, host: *Persea borbonia*: 1♀ emerged 18 Mar 1991 (UCMS); mines 22 Feb 1994, T. Dickel, DRD 1489, host: *Persea borbonia*: 1♂, 1♀ emerged 27 Feb 1994, 4♂, 2♀ emerged 4 Mar 1994, 1♂ emerged 7 Mar 1994, 1♂ emerged 8 Mar 1994 (USNM); 1 km W. Tamarind Hammock, 29 April 1992, T. Dickel, DRD 1088, host: *Persea borbonia*: 28 leaf mines, 1♂ emerged 7 May 1992, 1♂, 5♀ emerged 10–11 May 1992, 1♂ emerged 14 May 1992, (USNM); DRD 1020, 5 leafmines, 21 Nov 1991, D. Davis, host: *Persea borbonia*;DRD 1060.1, host: *Persea borbonia*, 17 Feb 1992, D. and S. Davis: 1♂ emerged (DOA) 12 Mar 1992, 1♂ emerged 24 Mar 1992 (USNM). VIRGINIA: Nansemond Co: Dismal Swamp near Lake Drummond: 1 leafmine, 7–8 Jul 1962, D. Davis, DRD 187, host: *Persea borbonia*; 1♂ with pupal exuvium, 8–10 June 1974, emerged 16 Jun 1974, D. and M. Davis, DRD 187.2, host: *Persea borbonia*; Virginia Beach Co: Seashore State Park [First Landing State Park]: 7 leafmines, 9 July 1962, D. Davis, DRD 187, host: *Persea borbonia* (USNM).

**Parasitoids**: Hymenoptera: Eulophidae: *Chrysocharis* sp., *Cirrospilus* sp., *Closterocerus* sp., *Elasmus* sp., *Horismenus* sp., *Sympiesis* sp.

**Flight** **Period**: We have had adults issue from our mine collections from southern Florida during September, December, February, March, April, May, and June; and in southern Virginia during June.

**Distribution**: This species has been found from Nansemond and Virginia Beach Counties, Virginia, USA, south along the lowland Atlantic coastal region to the Florida Everglades. Adults in the collections of the USNM, from avocado, some with associated mines and collected at various localities in Honduras, may also represent this species. No pupae were available for study and attempts to barcode two specimens were unsuccessful. Mines with associated pupae of what appear to be *hyperpersea* have also been intercepted on shipments of avocado within the United States from unspecified localities in Mexico. Some fluctuation in the northern limits of this leafminer may have occurred in recent years. As late as June 8–11, 1974, DRD and Mignon Davis found mines of *Phyllocnistis hypersersea* common on leaves of *Persea borbonia* within First Landing State Park and Dismal Swamp, Virginia. On March 14, 1992 and during August 1993 no mines could be found at First Landing State Park (Dismal Swamp was not visited in 1993). These localities have not been surveyed for leafminers since 1993.

**Etymology**: The specific name is derived from the Greek, *hyper* (above, over) and the generic plant name of its host, *Persea*, in reference to the characteristic leafmining habit of the larva on the upperside of the leaf. The specific epithet is a noun in the nominative singular.

#### Phyllocnistis subpersea

Davis and Wagner sp. n.

urn:lsid:zoobank.org:act:20FB0553-E021-401D-9475-9F0B06E115DA

http://species-id.net/wiki/Phyllocnistis_subpersea

Figs 2B 3B 5B 8A 13F 17A–E

##### Diagnosis:

*Phyllocnistis subpersea* is the phenotypicoutlier among the four *Persea*-feeding species that we treat here: the row of raised, broadened, black-tipped fringe scales along the tornal margin of the forewing is unique. The apical dot tends to be poorly developed. It is the only *Persea* feeder that consistently has a subbasal fuscous spot along the inner margin of the forewing (*Phyllocnistis hyperpersea* sometimes has fuscous scales in the subbasal area of the forewing, but these do not form a spot but rather extend as a diffuse patch to the wing base). The hind tarsomeres (especially segments 2–3) often bear orange to fuscous scaling that is somewhat more pronounced than that of the species that follow. The ductus bursae is broadly joined to corpus bursae. The frontal process of the pupa consists of a pair of stout conical spines arising near the apex, and a single, more subapical, strongly curved spine from the upper frons (Figs 12A–C).

##### Adult

(Fig. 2B): Length of forewing: 2.0 to 2.7 mm, although most measure between 2.4–2.6 mm.

*Head:* Frons shiny white, smooth glabrous, with subtle faint orange tints over vertex. Flagellomeres with faint orange luster above. Labial palpus white, short, roughened apically, length > height of eye; distal segment subequal to segment 2; segment 1 very short.

*Thorax:* Patagia and tegulae with stramineous to orange tints. Longitudinal fascia joining transverse fascia but weakened distad, edged with black scales above and below, with those below more consistently present distad. Transverse fascia leaves costal margin at 45° angle; lower arm where it leaves inner margin poorly defined, often fusing with diffuse subbasal patch of fuscous scales. Second costal fascia usually fusing with transverse fascia distally. Apical spot weakly developed, small, fuscous but not black in our material, composed of apices of a few to several scales. Apical strigulae vague and poorly differentiated. Black fringe scales about tornus broadened, conspicuously blackened apically, raised appreciably above plane of wing. Legs silvery white with exception of faint orange luster to dorsal and outer surfaces; foretibiae, foretarsi, and distal tarsomeres sometimes modestly darkened; hind tarsi with tarsomeres 2–4 with faint orange to fuscous tint.

*Abdomen:* Silvery white and unmarked.

*Male Genitalia* (Figs 17A–C): Similar to *Phyllocnistis hyperpersea* except valva curved slightly dorsad; relatively shorter, ~ 1.6× length of vinculum; basal apodeme of valva directed slightly caudad in repose.

*Female Genitalia* (Figs 17D–E): Similar to *Phyllocnistis hyperpersea* except ductus bursae slightly shorter, ~ 2.2× length of papillae anales and gradually enlarging to moderately slender, elliptical corpus bursae; ductus seminalis ~ 1.8× length of corpus bursae.

##### Larva

(Figs 5B): Hypermetamorphic; early instars with highly modified, depressed body for sapfeeding. Final instar non-feeding, with all mouthparts reduced or absent except for functional spinneret.

**Figure 8.**
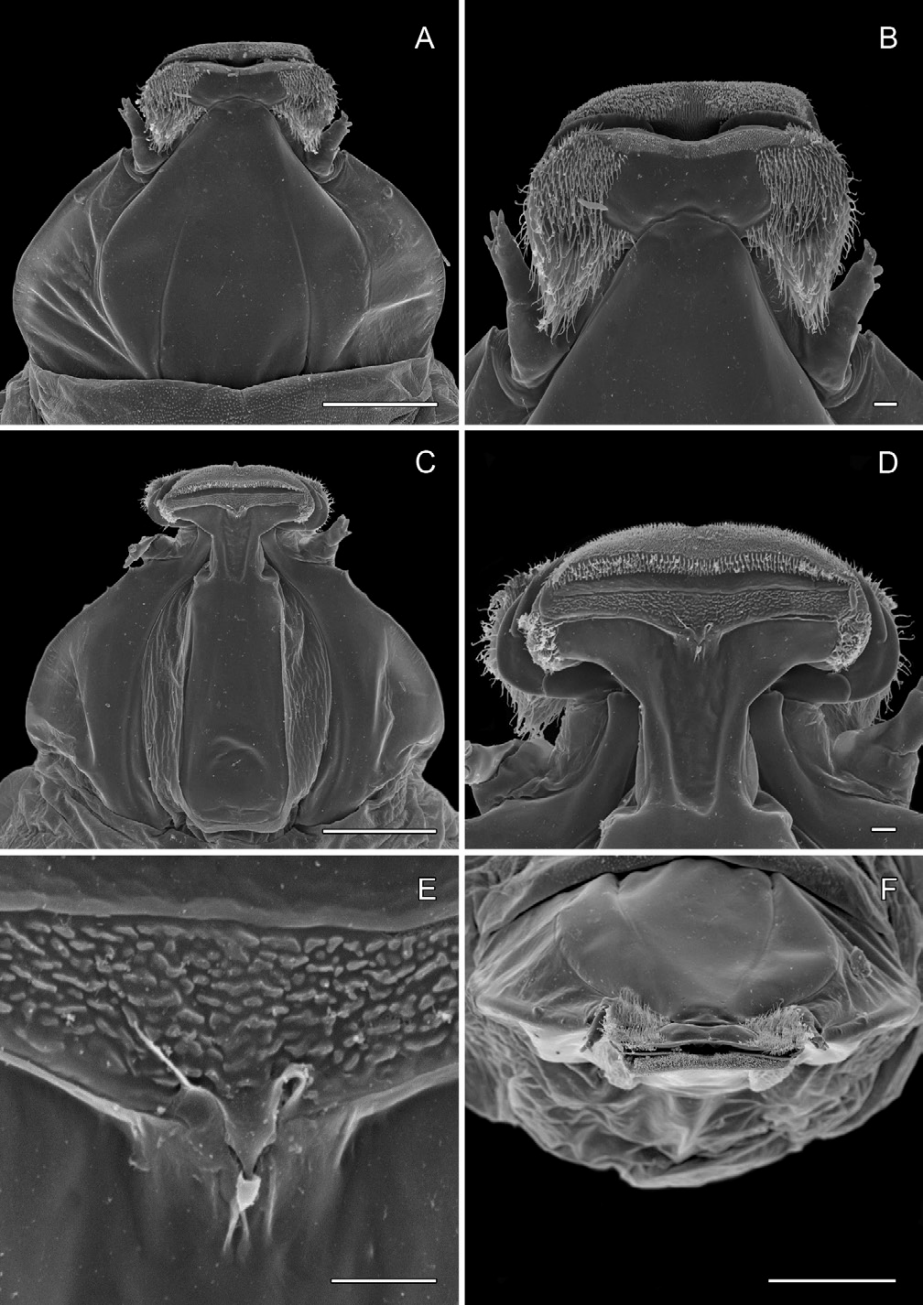
*Phyllocnistis subpersea* sp. n. head of sapfeeding larva, late instar **A** Dorsal view (100 µm) **B **Detail of labrum and antennae in **A** (10 µm) **C** Ventral view (100 µm) **D** Detail of labium in **C** (10 µm) **E** Spinneret (10 µm) **F** Anterior view of head (100 µm). (Length of bar scales shown in parentheses.)

**Figure 9.**
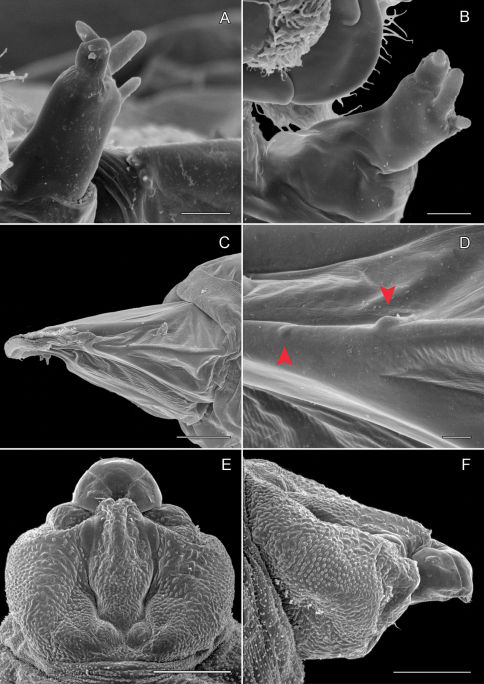
*Phyllocnistis subpersea* sp. n. head of larva. **A–D** Head of sapfeeding larva, late instar. **A** Antenna, dorsal view (10 µm) **B** Antenna, ventral view (10 µm) **C** Lateral view (100 µm) **D** Stemmata (indicated by arrows; 10 µm) **E–F** Head of last instar (spinning) larva: **E** Dorsal view (111 µm) **F** Lateral view (100 µm). (Length of bar scales shown in parentheses.)

*Sapfeeding instar* (Figs 5B, 8A–9D): Length of largest larva examined ~ 4.7 mm. Head prognathous, greatly depressed; primary setae either lost or reduced; two stemmata present laterally in a single, well-spaced horizontal alignment; antenna 3-segmented (Figs 9A–B), second segment more slender than first, with 2 moderately stout and 1 short sensillae; third segment less that 1/3 the length of second, with single, apical sensillum basiconicum; labrum with well-developed, densely spinose, lateral lobes; anterior lateral margins rounded; posterior lateral margins extended caudally as triangular lobes; anterior ventral margin densely spinose. Labium also with well-developed lateral lobes; rugose band of cuticle extending across anterior ventral margin (Fig. 8D). Spinneret rudimentary, with narrow, acute extension of cuticle largely covering aperture (Fig. 8E). Legs and prolegs absent. Last (9+10th) segment of abdomen with pair of caudal processes ~ 0.75× length of entire segment (Fig. 5B).

**Figure 10.**
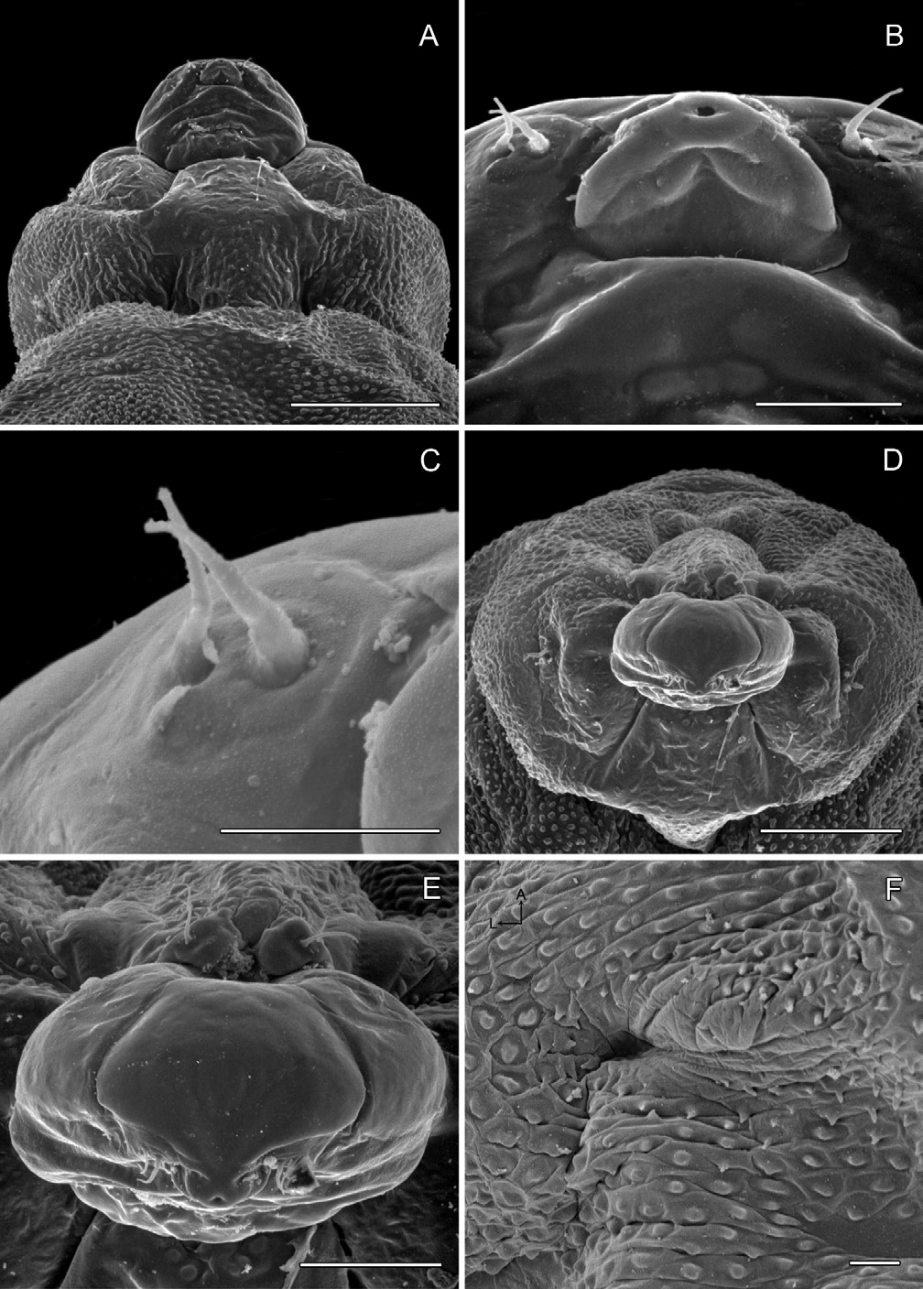
*Phyllocnistis subpersea* sp. n. last instar (spinning) larva. **A** Head ventral view (111 µm) **B** Detail of spinneret in **A** (17.6 µm) **C** Detail of maxilla in **B** (10 µm) **D** Anterior view of head (100 µm) **E **Detail of anterior trophic lobe in **D** (43 µm) **F** Left ambulatory callus on abdominal sternum 6 (10 µm). (Length of bar scales shown in parentheses.)

**Figure 11.**
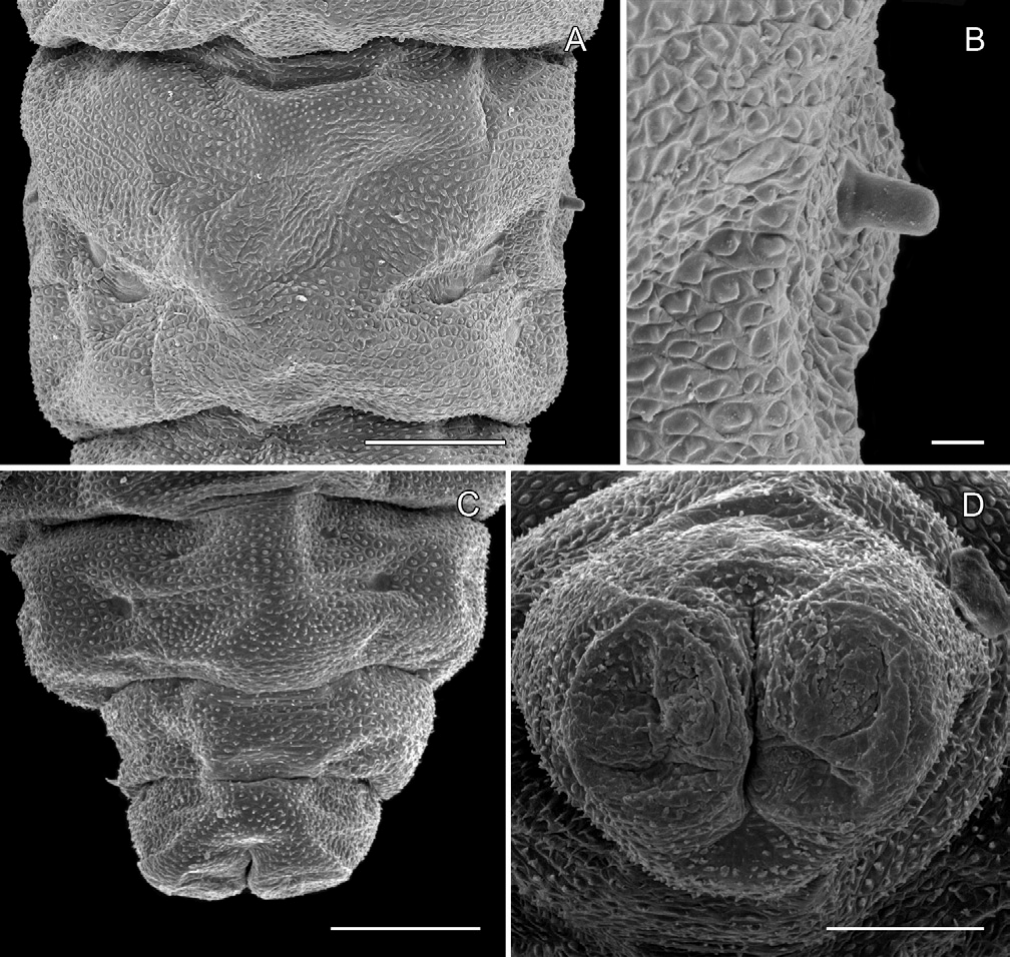
*Phyllocnistis subpersea* sp. n. last instar (spinning) larva. **A** Abdominal tergum 7 (100 µm) **B** Detail of seta SD1 in **A** (10 µm) **C** Abdominal sterna 7–10 (100 µm) **D** Caudal view of abdomen (60 µm). (Length of bar scales shown in parentheses.)

*Spinning (last) instar* (Figs 9E-11D): Body cylindrical, with all appendages and setae greatly reduced; integument finely tuberculate; length of largest larva examined ~ 4.1 mm. Head capsule weakly sclerotized, slightly broader than long; integument finely tuberculate; trophic region extended anteriorad as well-defined lobe; stemmata absent; antenna 2-segmented; first (basal) segment greatly reduced, nearly flush with head capsule, with 2 sensilla basiconica and 1 sensillum chaeticum; apical segment consisting of single sensillum basiconicum. Trophic lobe (Figs 10A–E) with relatively broad but shortened spinneret with simple, terminal opening and rudimentary maxilla; maxilla flush with head capsule and represented by pair of moderately long and one very short sensilla chaetica. Thoracic legs absent, with only indistinct paired ventral callosities on T1–3. Abdomen without prolegs; paired ventral callosities present on A3–6 (Fig. 10F); seta SD1 much larger than other setae, relatively stout and short (Figs 11A–B); A10 truncate, without caudal lobes or callosities (Fig. 11C–D).

##### Larval Mine

(Figs 3B): Similar to that described for *Phyllocnistis longipalpa*. A long, slender, serpentine gallery, containing a dark, median frass trail, on the underside or occasionally the upperside of the leaf, with pupation occurring in a slightly enlarged, elliptical chamber at the mine terminus along a leaf edge. The egg is deposited away from the midrib, usually on the lower side of the leaf. Mine width increases from ~ 0.3 mm broad to a maximum width of ~ 2–2.5 mm; width of the frass trail is usually about half the mine width.

##### Pupa

**Figure 12.**
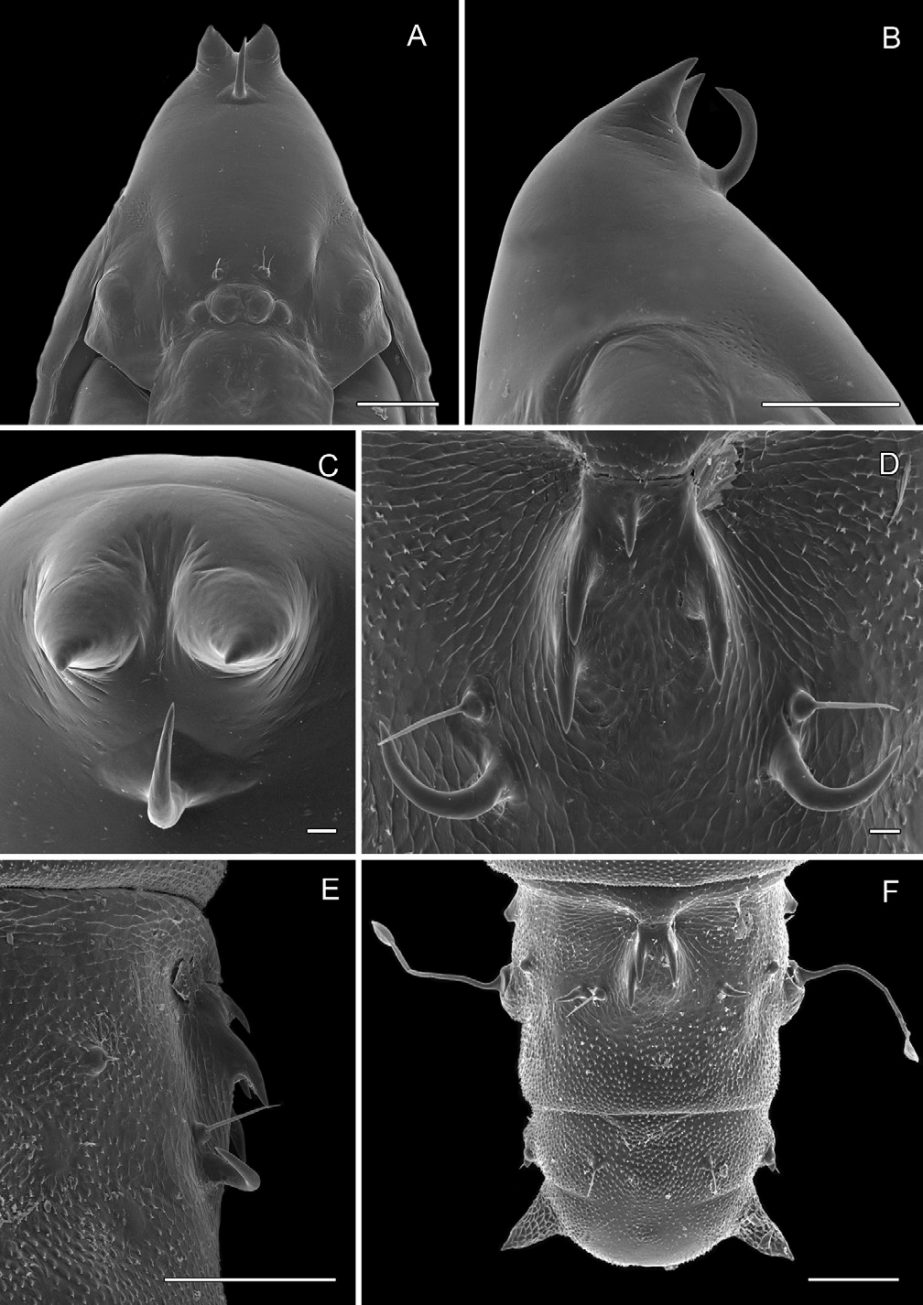
*Phyllocnistis subpersea* sp. n. pupa. **A** Head, ventral view (100 µm) **B** Head, lateral view (100 µm) **C** Head, anterior view (10 µm) **D** Dorsal spines of abdominal tergum 6 (10 µm) **E** Lateral view of **D** (100 µm) **F** Abdominal terga 7–10 (100 µm). (Length of bar scales shown in parentheses.)

**Figure 13.**
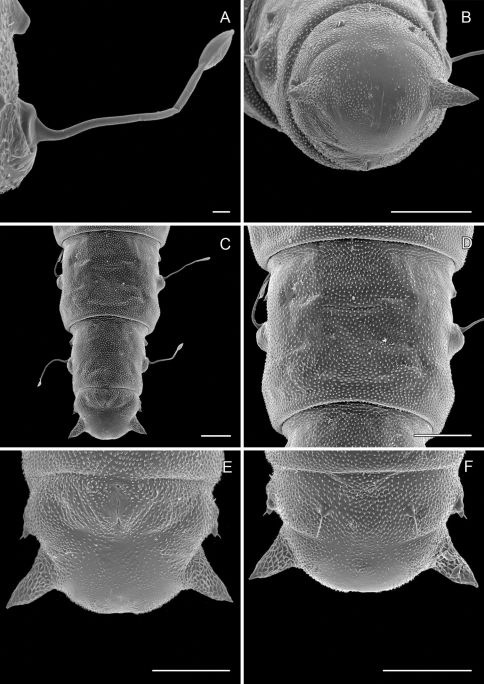
*Phyllocnistis subpersea* sp. n. pupa. **A** Lateral seta of abdominal segment 6 (10 µm) **B** Caudal end of abdomen (100 µm) **C** Abdominal sterna 6–10 (100 µm) **D** Scattered spinules of abdominal sternum 6, (100 µm) **E** Abdominal sterna 8–10 (100 µm) **F** Abdominal terga 8–10 (100 µm). (Length of bar scales shown in parentheses.)

(Figs 12A–13F): Similar to *Phyllocnistis hyperpersea* except: length of largest pupa 3.2 mm. Vertex with pair of stout conical, spines arising near apex, and single, slender, more subapical, strongly recurved spine from upper frons (Figs 12A–C). Abdomen with 6 pairs of small, sclerotized, oval, median pits near anterior margins of terga 2–7; each sclerotized pit giving rise to 2 columns of low, strongly recurved spines, relatively larger than in *hyperpersea* and fewer in number, arranged in about 1–3 ranks (Figs 12D–E); pair of slightly larger, strongly recurved spines immediately lateral to caudal end of median cluster and nearly contiguous to seta D1; A2–7 with SD1 setae greatly lengthened, apices spatulate (Fig. 13A); sternum A6 with spinules evenly scattered over surface as in *hyperpersea* (Fig. 13D); A10 with pair of relatively large, stout, caudal processes directed mostly laterally (Figs 13E–F).

##### Host:

*Persea borbonia* (L.) Spreng.

##### Type Material:

Holotype: ♂, USA: FLORIDA: Dade Co: Everglades National Park, Long Pine Key, 26°24'N, 80°41'W, mine 21 Feb 1992, emerged 28 Feb 1992, D. and S. Davis, DRD 1061, host: *Persea borbonia*, (USNM). Paratypes: USA: FLORIDA: Dade Co: Everglades National Park: Long Pine Key, 26°24'N, 80°41'W, mines 21 Feb 1992, D. and S. Davis, DRD 1061, host: *Persea borbonia*, 1 ♀ emerged 22 Feb 1992, BOLD ID: RDOPO391-09, 1♂ emerged 26 Feb 1992, USNM slide 31632; 1♂ emerged 28 Feb 1992; 1♂ emerged 1 Mar 1992, USNM slide 31634; 2♂, 2♀ emerged 3 Mar 1992, 1♂, BOLD ID: RDOPO388-09, 2♀ emerged 6 Mar 1992, 1♀ emerged 10 Mar 1992 (USNM). Pa-hay-okee Overlook, 26°27'N, 80°47'W: mines 16 Apr 1995, D., M., and S. Davis, DRD 1624.1, host: *Persea borbonia*: 2♂, 4♀ emerged 28 Apr 1995, BOLD ID: RDOPO389-09. Monroe Co: Big Cypress National Preserve, Loop Road near Tamarind Hammock, 26°27'N, 80°31'30"W, mines 13 Mar 1991, D. L. Wagner and D. R. Davis, DLW Lot: 91C139, host: *Persea borbonia*: 8♂, 9♀, 2 unsexed emerged 15–25 Mar 1991 (UCMS); mines 21 Nov 1991, D. L. Wagner and D. R. Davis, DLW Lot: 91L35, host: *Persea borbonia*: 4♂, 8♀, 4 unsexed, emerged 25 Nov-4 Dec 1991 (UCMS); mines 22 Feb 1994, D. Davis, DRD 1490, host: *Persea borbonia*: 1♀ emerged 7 Mar 1994, BOLD ID: RDOPO392-09, 1♂ emerged 8 Mar 1994; 3♂, 2♀ emerged 4 Mar 1994; mines 25 Mar 1994, T. Dickel, DRD 1490.1, host: *Persea borbonia*: 2♀, emerged 4 Apr 1994, (USNM). Loop Road, Tamarind Hammock, 25°27'N, 81°16'W: 11 Apr 1995, D., M., and S. Davis, DRD 1624, host: *Persea borbonia*, 2 ♂, emerged 11 Apr, 1995, BOLD ID: RDOPO390-09 (USNM).

**Parasitoids**: Hymenoptera: Eulophidae: *Cirrospilus* sp., *Galeopsomyia* sp., *Horismenus* sp.

**Flight** **Period**: Adults (from recently collected mines) have emerged from February 22 to April 11 in Florida.

**Distribution**: At least Dade and Monroe Counties, Florida. We have found mines of what appear to be this species on *Persea borbonia* as far north as the Green Swamp in coastal South Carolina.

**Etymology**: The specific name is derived from the Greek, *sub* (under) and the generic plant name of its host, *Persea*, in reference to the characteristic leafmining habit of the larva usually on the underside of the leaf. The specific epithet is a noun in the nominative singular.

#### Phyllocnistis longipalpa

Davis and Wagner sp. n.

urn:lsid:zoobank.org:act:4566411E-4481-424B-9543-9F7383ACEB35

http://species-id.net/wiki/Phyllocnistis_longipalpa

Figs 2C 18A–E.

##### Diagnosis:

*Phyllocnistis longipalpa* can be distinguished from other *Persea*-feeding *Phyllocnistis* in the southeastern United States, by its long, slightly upcurved labial palpi (> height of head). The apical spot is poorly developed, which distinguishes it from *hyperpersae*. It lacks the run of raised black scales from the forewing tornus characteristic of *Phyllocnistis subpersea*.

##### Adult

(Fig. 2C): Length of forewing: 2.3 to 2.6 mm.

*Head:* Frons shiny white, smooth with subtle faint orange tints over vertex. Flagellomeres with faint orange luster above. Labial palpus white, long, 1.2× height of head, slightly upcurved; basal segment subequal to segments 2 + 3.

*Thorax:* Patagia and tegulae with subtle, silvery, stramineous to orange tints. Longitudinal fascia joining transverse fascia, edged with black scales above and below, with those below more consistently present distad. Transverse fascia leaves costal margin at 45° angle; proximal edge of transverse fascia where it leaves the inner margin vague, composed of 2–3 rows of dark scales. Second costal fascia fusing with transverse fascia distally. Apical spot poorly differentiated; likewise apical strigulae vague and poorly developed. Black fringe scales about tornus little broadened and not conspicuously elevated above plane of wing. Legs essentially silvery white and unmarked with exception of faint orange luster to dorsal and outer surfaces of foretibiae and foretarsi; distal tarsomeres sometimes modestly darkened.

*Abdomen:* Silvery white and unmarked.

*Male Genitalia* (Figs 18 A–C): Similar to *Phyllocnistis hyperpersea* and *subpersea* except apex of valva not evenly rounded, instead more oblique and extended dorsad; total length of valva ~ 2.0× length of vinculum; basal apodemes of valva less divergent than in other species, with ventral apodeme strongly curved (Fig. 18B). Aedeagus ~ 0.65× length of valva.

*Female Genitalia* (Figs 18 D–E): Similar to *Phyllocnistis perseafolia*, with ductus bursae long, ~ 6.5× length of papillae anales and terminating near caudal end of corpus bursae; corpus bursae elongate-ovoid, enlarged, ~ 0.6× length of elongate ductus bursae; ductus seminalis ~ 2.4× length of corpus bursae.

##### Larva and pupa:

Not examined.

##### Larval Mine:

Similar to that described for *Phyllocnistis subpersea*. A long, slender, serpentine gallery, containing a dark, narrow, median frass trail, present on the underside of the leaf, with pupation occurring in a slightly enlarged, elliptical chamber at the mine terminus along the leaf edge.

##### Host:

*Persea borbonia* (L.) Spreng.

##### Type Material:

Holotype: ♂, USA: FLORIDA: Dade Co: Everglades National Park: Pa-hay-okee Overlook, 26°27'N, 80°47'W: mines 12 Apr 1998, emerged 29 Apr 1998, D., M., and S. Davis, DRD 2135.1, host: *Persea borbonia*, (USNM). Paratypes : USA: Same data as holotype except: 1 ♂, emerged 14 Apr 1998; 3 ♂, 2 ♀ emerged 19 Apr 1998, ♀ slides 34206, 34209; 2 ♂, 1 ♀ emerged 29 Apr 1998, ♂ slides 34176, 34178, ♀ slide 34177, BOLD ID: RDOPO401-09 (USNM). Cheika [Recreation Area], NW Homestead: mines 12 Apr 1998, D., M., and S. Davis, DRD 2135, 1♀ emerged 14 Apr 1998; 2♂ emerged 19 Apr 1998, BOLD ID: RDOPO402-09 (USNM). Monroe Co: Loop Road, Tamarind Hammock, 25°27'N, 81°16'W: mine 11 Apr 1995, D., M., and S. Davis, DRD 1624, host: *Persea borbonia*, 1 ♂, emerged 11 Apr, 1995, BOLD ID: RDOPO400-09 (USNM). The holotype is provisionally deposited at the USNM, Washington, D.C., pending mutual resolution and agreement with the National Park Service regarding specimen deposition.

##### Parasitoids: 

Unknown.

##### Flight Period:

Adults have emerged in April in southern Florida.

##### Distribution:

Known only from the Everglades National Park, Dade County, and along the Loop Road near Tamarind Hammock, Monroe County, Florida.

##### Etymology:

The specific name is derived from the Latin *longus* (long) and *palpus* (feeler), in reference to the elongate labial palpi, which are diagnostic for this species. The specific epithet is a noun in the nominative singular.

##### Remarks:

We initially “discovered” *Phyllocnistis longipalpa* intermixed among our series of *Phyllocnistis subpersea* in 2009. As noted in the diagnosis, adults are reliably distinguished from that species by their longer labial palpi, the absence of the numerous, broad, raised tornal scales, and absence of the fuscous subbasal spot along the inner margin on the forewing which occurs in most *subpersea.* The larvae form serpentine mines on the undersides of new leaves, similar to those of *Phyllocnistis subpersea*.

#### Phyllocnistis perseafolia

Davis and Wagner sp. n.

urn:lsid:zoobank.org:act:D4F0984F-4A66-4FF6-AB1A-9C716E326361

http://species-id.net/wiki/Phyllocnistis_perseafolia

Figs 2D 3C 4A–B 14A 15D 19A–E.

##### Diagnosis:

*Phyllocnistis perseafolia* is the largest of the *Persea*-feeding species: forewing lengths typically exceed 2.6 mm, with that of intact specimens often reaching lengths of 2.9 or more mm. The well-developed black apical dot distinguishes *Phyllocnistis perseafolia* from all but *Phyllocnistis hyperpersea*. The forewing is the palest of the four *Phyllocnistis* described here: the black scaling--in the subbasal and tornal areas, as well as that which edges the longitudinal and transverse fascia--is reduced relative to the other species described here. The transverse fascia is often interrupted through the center of the forewing because the arms are so strongly angled outward that they may not meet; likewise the longitudinal fascia frequently does not join the transverse fascia in *persaefolia* for the same reason.

##### Adult

(Fig. 2D): Length of forewing: 2.6–3.2 mm.

*Head:* Frons shiny white, smooth glabrous. Flagellomeres with faint orange luster above but becoming smoky toward apex. Labial palpus white, long, slender, subequal to height of head, slightly upcurved; basal segment subequal to segments 2 + 3.

*Thorax:*Scaling of patagia and tegulae damaged. Longitudinal fascia ending before transverse fascia; anterior side ill-defined with orange scales often reaching to costa, especially towards base of wing; lower side straight and clearly delineated; fuscous scales, if present, only along lower edge. Transverse fascia edged inwardly and outwardly with black scales; upper arm leaving costal margin at 30–35° angle, with distal reach curving toward apical dot; often interrupted through cell; arm of transverse fasciae from inner margin more strongly edged with black along outer edge; proximal edge of fascia where it leaves the inner margin vague, with faint dark scaling. Second costal fascia ill-defined, with little black scaling, sometimes conjoined with transverse fascia. Three costal and three apical strigulae modestly differentiated. Apical spot well developed. Black fringe scales about tornus reduced in extent, many replaced with smoky orange fringe scales; none raised appreciably above plane of wing. Legs essentially silvery white and unmarked with exception of faint orange luster to dorsal and outer surfaces foretibiae and foretarsi and distal tarsomeres sometimes with smoky overscaling.

*Abdomen:* Silvery white and unmarked.

*Male Genitalia* (Figs 19A–C): Similar to *Phyllocnistis hyperpersea*, with approximately straight valva, except valva relatively shorter, ~ 1.6× length of vinculum; basal apodemes of valva more widely divergent than in other species, with ventral apodeme approximately straight (Fig. 19B).

*Female Genitalia* (Figs 19D–E): Similar to *Phyllocnistis longipalpa*,with ductus bursae joining corpus bursae near caudal end; corpus bursae elongate-ovoid, enlarged, ~ 0.5× length of elongate ductus bursae; ductus seminalis ~ 2.25× length of corpus bursae.

##### Larva:

Not examined.

##### Larval Mine

(Figs 3C, 4A–B): Similar to that described for *Phyllocnistis subpersea*. A long, slender, serpentine gallery, containing a dark, narrow, median frass trail, present on either the underside or upperside of the leaf, with pupation occurring in a slightly enlarged, elliptical chamber at the mine terminus along the leaf edge. Serpentine mines of possibly this species have also been observed by Francisco Posada on avocado fruit at the type locality.

##### Pupa

**Figure 14.**
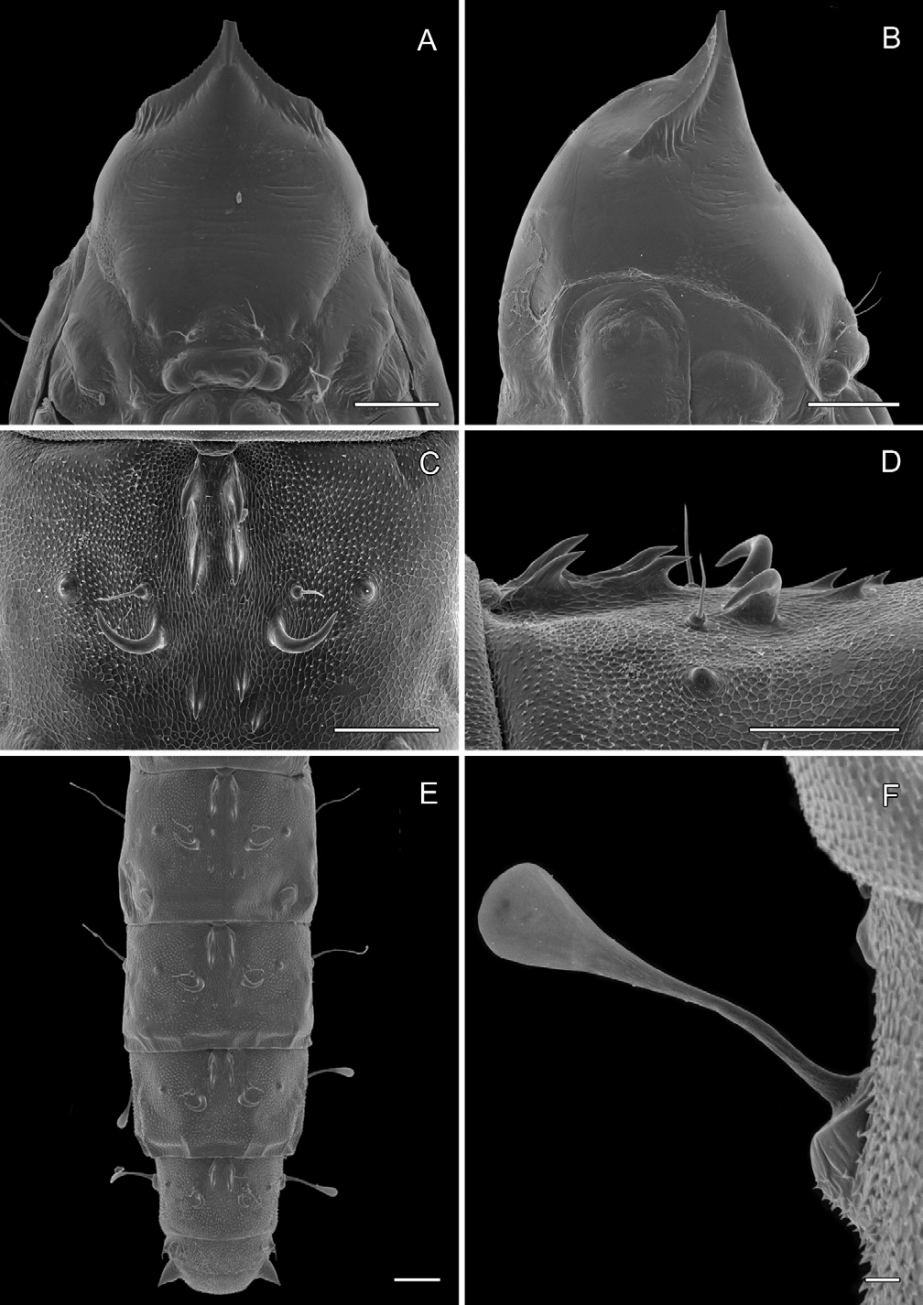
*Phyllocnistis perseafolia* sp. n. pupa. **A** Head, ventral view (176 µm) **B** Head, lateral view (200 µm) **C** Dorsal spines of abdominal tergum 5 (76 µm) **D** Lateral view of **C** (60 µm) **E** Abdominal terga 4–10 (100 µm) **F** Lateral seta of abdominal segment 6 (10 µm). (Length of bar scales shown in parentheses.)

**Figure 15.**
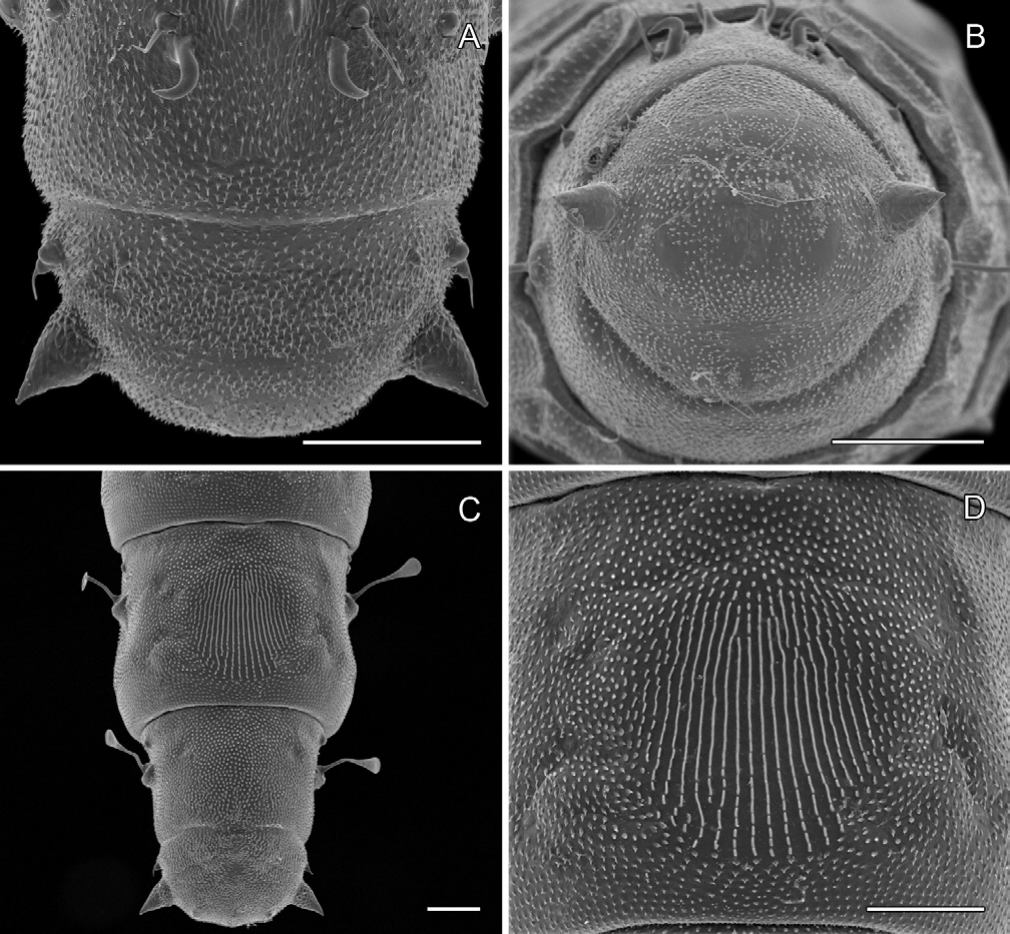
*Phyllocnistis perseafolia* sp. n. pupa. **A** Abdominal terga 7–10 (100 µm) **B** Caudal end of abdomen (100 µm) **C** Abdominal sterna 6–10 (100 µm) **D** Spinules of sternum 6 in longitudinal rows (100 µm). (Length of bar scales shown in parentheses.)

(Figs 14A-15D): Similar to *Phyllocnistis hyperpersea* except: Length of largest pupa 3.4 mm. Vertex similar to that of *Phyllocnistis vitegenella* Clemens in possessing single, large apical spine (tip of spine broken in all 3 pupae examined) with minutely serrated, low ridge descending laterally from spine (Figs 14A–B). Abdomen with apices of greatly lengthened SD1 seta on A2–7 spatulate; mid-dorsal cluster of spines on abdominal terga 2–7 (Figs 14C–D) with median series of low, strongly recurved spines relatively larger than in *hyperpersea* and fewer in number, arranged instead in 2 short columns as in *subpersea* in about 2 ranks; 3–4 smaller, scattered spines immediately caudad to larger, median spine rows; pair of slightly larger, strongly recurved spines immediately lateral to caudal end of median cluster and nearly contiguous to D1 seta; sternum A6 with spinules arranged in ~ 20 longitudinal ridge-like rows (Fig. 15D); A10 with pair of relatively large, stout, caudal processes directed mostly laterally (Figs 15A–C) as in *subpersea*.

**Figure 16.**
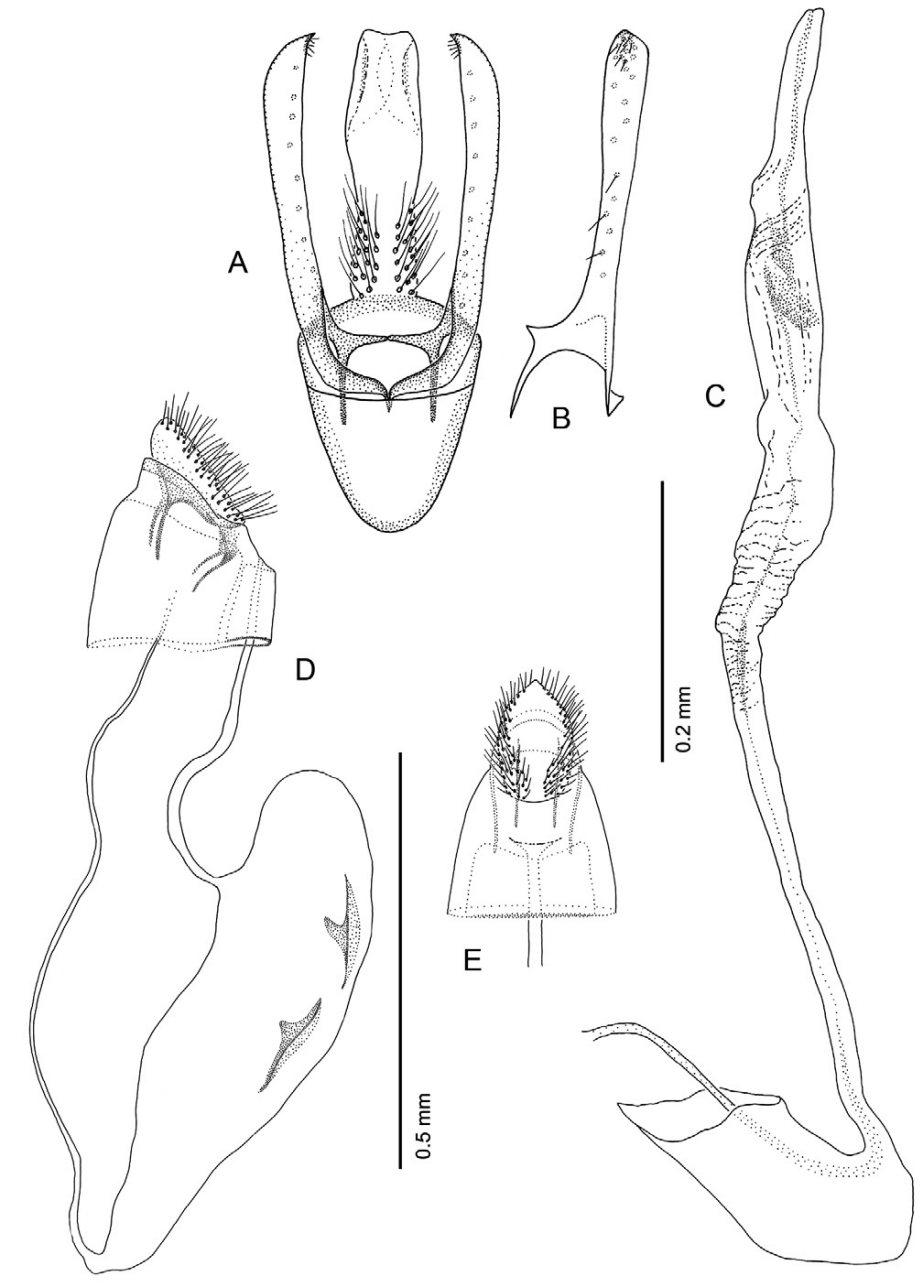
*Phyllocnistis hyperpersea* sp. n. genitalia. **A** Male, ventral view **B** Mesal view of valva **C** Aedeagus **D** Female, lateral view **E** Ventral view of **D** segments 7–10.

**Figure 17.**
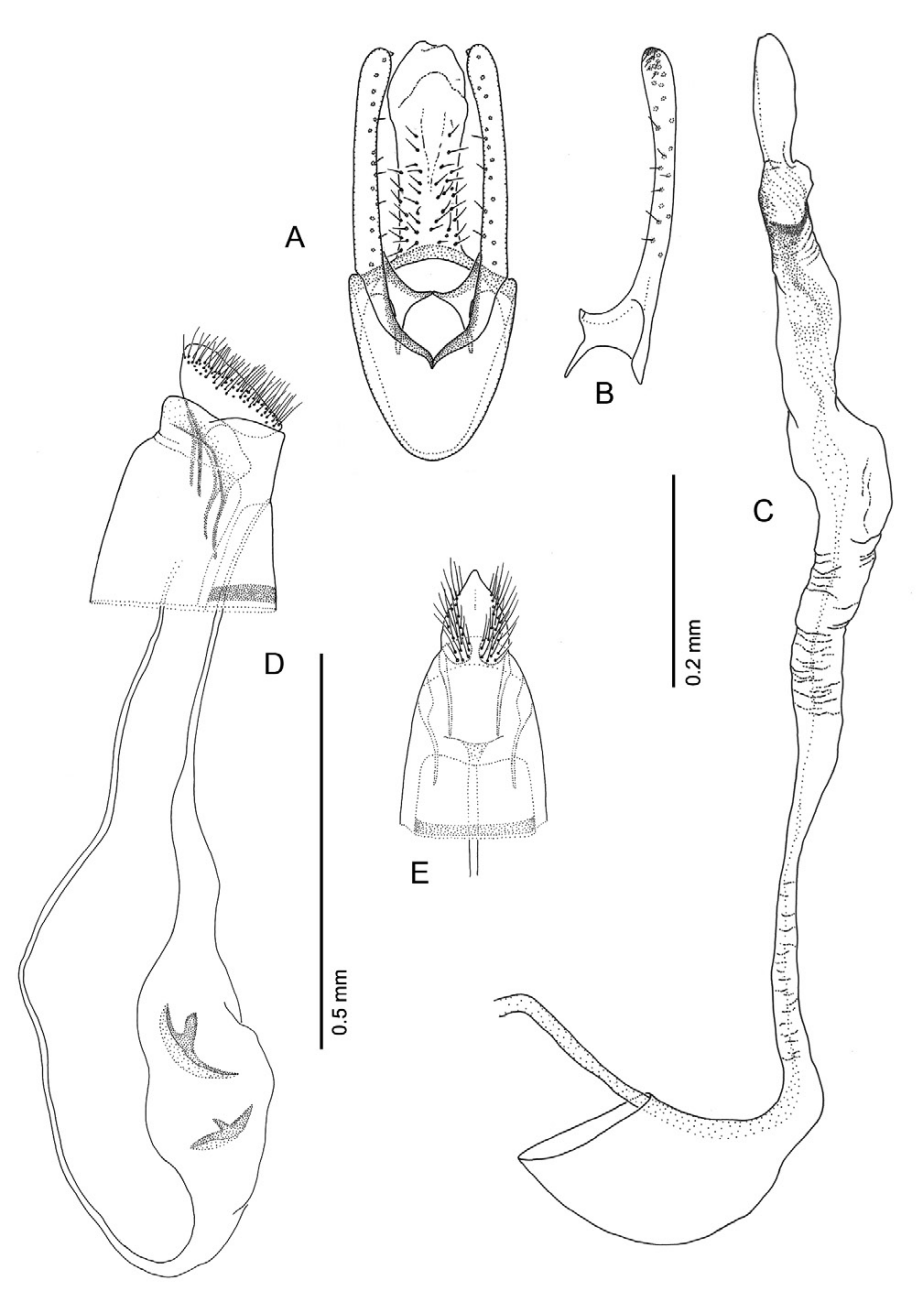
*Phyllocnistis subpersea* sp. n. genitalia. **A** Male, ventral view **B** Mesal view of valva **C** Aedeagus **D** Female, lateral view **E** Ventral view of **D** segments 7–10.

**Figure 18.**
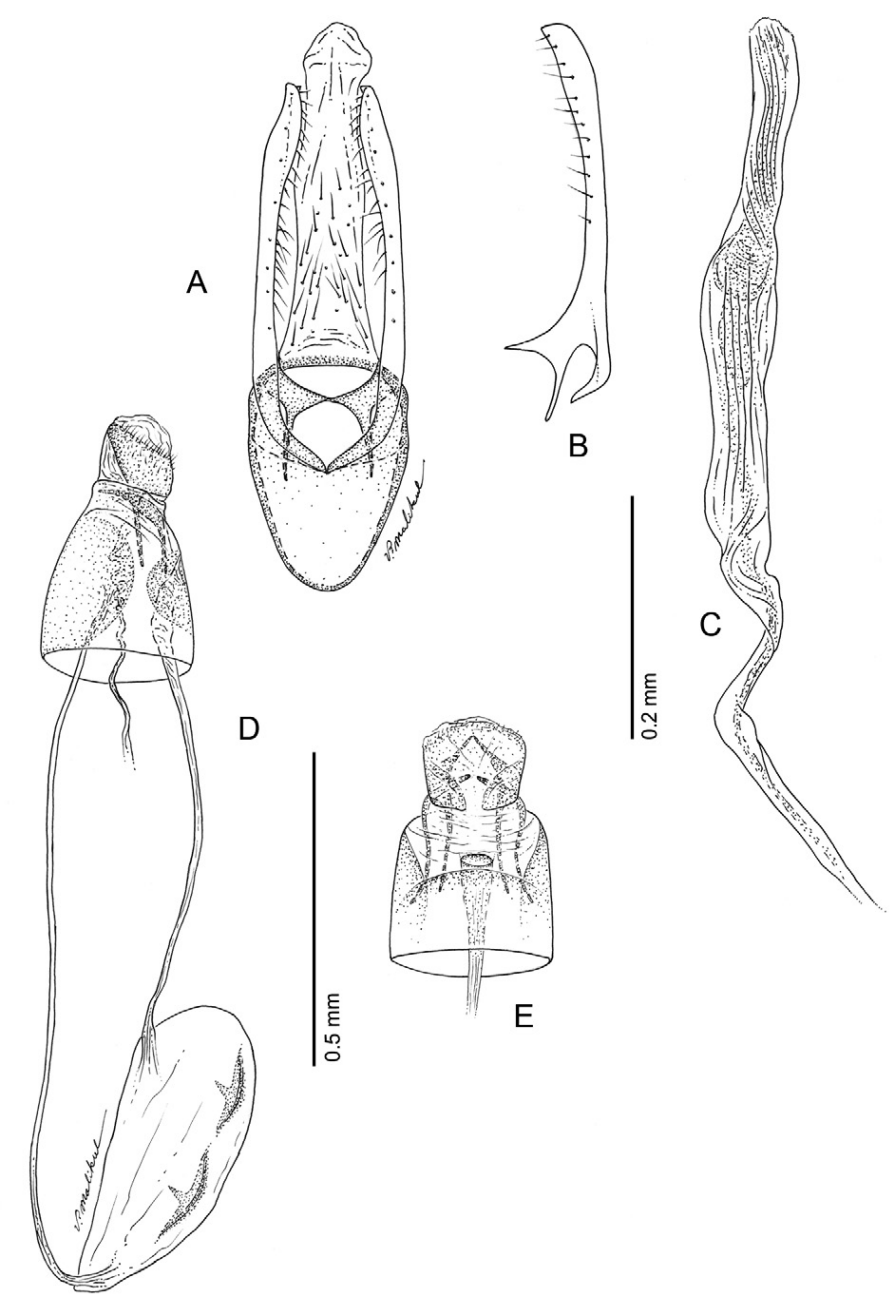
*Phyllocnistis longipalpa* sp. n. genitalia. **A** Male, ventral view **B** Mesal view of valva **C** Aedeagus **D** Female, lateral view **E** Ventral view of **D** segments 7–10.

**Figure 19.**
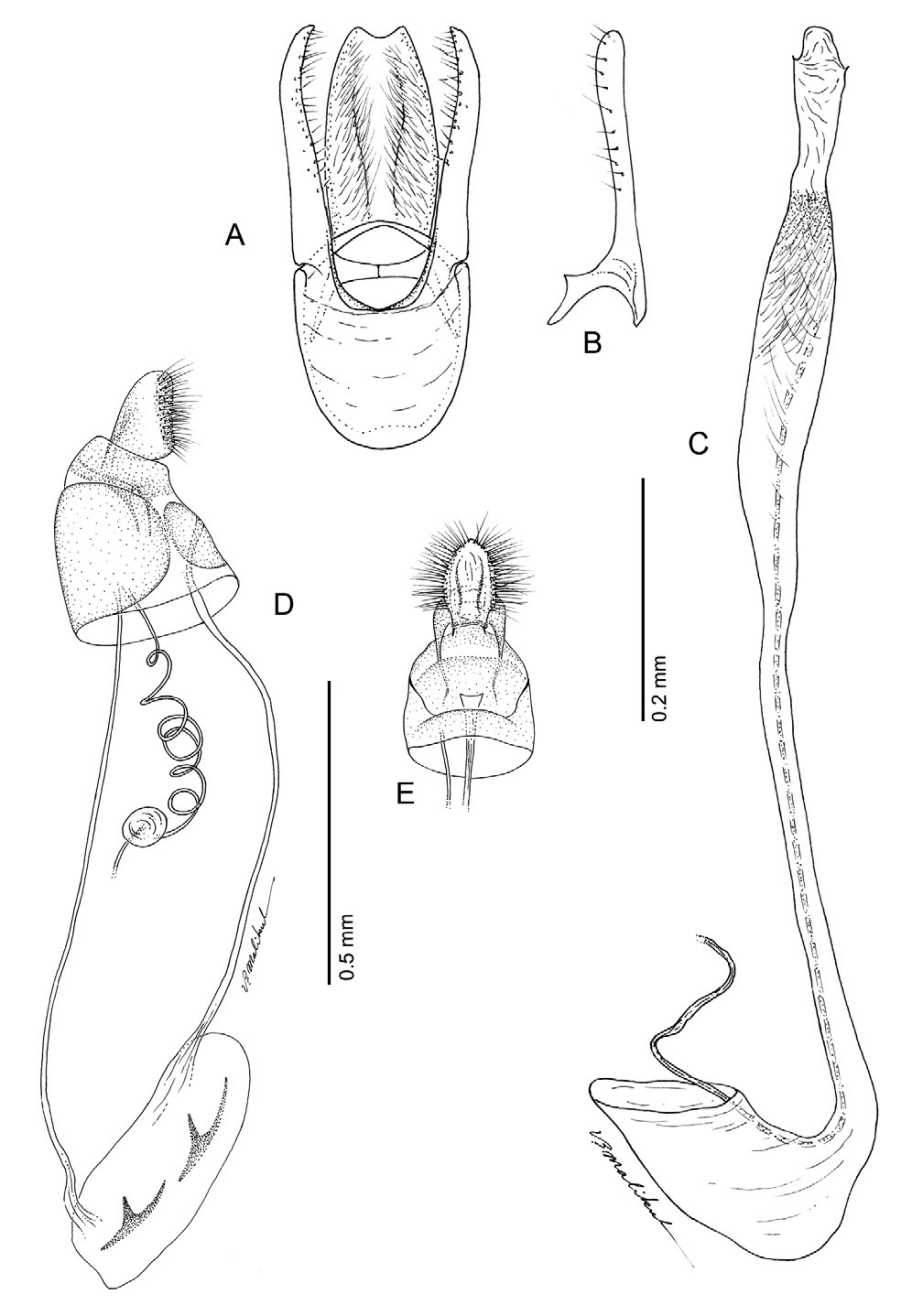
*Phyllocnistis perseafolia* sp. n. genitalia. **A** Male, ventral view **B** Mesal view of valva **C** Aedeagus **D** Female, lateral view **E** Ventral view of **D** segments 7–10.

##### Host:

*Persea americana* Mill., variety Hass.

##### Type Material:

Holotype: ♂, COLOMBIA: Caldas Department, Villamaria, April 2008, Francisco Posada, reared from Avocado, *Persea americana*, variety Hass, slide USNM 34075 (USNM). Paratypes: COLOMBIA: Same locality and data as holotype: 9 ♂, slides USNM 34078, 8 ♀, slides USNM 34076, 34077, BOLD ID: RDOPO393-10, RDOPO394-10; 5 pupae, USNM slide 34072 (UNCM, USNM).

##### Parasitoids:

Unknown.

##### Flight Period:

Adults have emerged in April in Colombia.

##### Distribution:

Currently reported only from the type locality in the Department of Caldas, west-central Colombia, but probably widespread over northern South America wherever avocado is cultivated.

##### Etymology:

The specific name is derived from the generic plant name of its host, *Persea* and the Latin, *folium* (leaf), in reference to its leafmining habit. The specific epithet is a noun in the nominative singular.

##### Remarks:

All adults examined were received unpinned, unspread, and slightly damaged to the extent that we are uncertain of some scaling characters. The apex of the large frontal process was broken in all 3 pupae available for study. A fragment of one spine remaining in a vial with a pupa of *perseafolia* was observed to be slightly recurved, but not to the extent observed in pupae of the North American *Phyllocnistis vitegenella*. The spatulate apex of abdominal SD1 setae of *perseafolia* (Fig. 14F) is notable in being the broadest of the three species examined.

One other species of *Phyllocnistis*, *Phyllocnistis aurilinea* (*auriinea* [sic]) Zeller, has been described from Colombia (Bogotá). However, that species mines the leaves of a distinctly different host in the family Ericaceae, “*Uva camarona*” (Zeller 1877), (probably *Macleania rupestris* A. C. Smith, according to W. and J. De Prins 2011), Because larvae of *Phyllocnistis* and related gracillariids are known to be stenophagous, *Phyllocnistis aurilinea* is believed to represent a different species from *Phyllocnistis perseafolia*, whose larvae are leafminers in Lauraceae.

## Supplementary Material

XML Treatment for Phyllocnistis hyperpersea

XML Treatment for Phyllocnistis subpersea

XML Treatment for Phyllocnistis longipalpa

XML Treatment for Phyllocnistis perseafolia
